# Case Report: Scar contracture caused by ruptured subcutaneous ganglion cyst misdiagnosed as Dupuytren's disease: diagnostic reflections on a rare case

**DOI:** 10.3389/fsurg.2026.1744274

**Published:** 2026-05-29

**Authors:** Kai Jiang, Gang Li, Liang Ma, Xue-zhen Liang, Yi-fa Rong, Jun Zhang

**Affiliations:** 1 The First College of Clinical Medicine, Shandong University of Traditional Chinese Medicine, Shandong, China; 2 Orthopaedic Microsurgery, Affiliated Hospital of Shandong University of Traditional Chinese Medicine, Shandong, China

**Keywords:** bleomycin, Dupuytren’s disease, ganglion cyst, Hodgkin’s lymphoma, scar contracture

## Abstract

This study reports a case of scar contracture on the volar side of the left little finger secondary to ruptured ganglion cyst in a 26-year-old female patient, which was misdiagnosed as Dupuytren's disease. The patient had a medical history of Hodgkin's lymphoma and received bleomycin chemotherapy, which may have accelerated the fibrotic process. Intraoperative findings were inconsistent with the preoperative diagnosis, and the final diagnosis was confirmed by pathological examination. Complete surgical resection successfully relieved the contracture. This case suggests that the etiology of subcutaneous scar contracture is complex. Comprehensive evaluation combined with patient medical history, medication history and pathological results is essential to achieve accurate diagnosis.

## Introduction

Dupuytren's disease is a chronic progressive disease, usually manifested as subcutaneous nodules and cords beneath the palmar skin, which may eventually result in finger flexion contracture (especially metacarpophalangeal joints and proximal interphalangeal joints), thus affecting hand function (1, 2). Dupuytren's disease shows prominent ethnic, age and gender clustering: its prevalence reaches approximately 30% in males aged over 60 in Northern Europe, while it is rare among people of African descent, and onset in young individuals is highly atypical (3). After selective aponeurectomy, the 5-year recurrence rate of the disease ranges from 20% to 40% (4). Bleomycin is a chemotherapeutic agent approved for the treatment of various malignant tumors. It can upregulate collagen synthesis in fibroblasts and activate the TGF-β pro-fibrotic signaling pathway, while also inducing severe adverse reactions such as organ fibrosis and flagellate dermatitis (5, 6). This study reports a female patient with ruptured ganglion cyst on the volar side of the little finger. The patient had a previous history of Hodgkin's lymphoma and developed little finger contracture deformity accompanied by obvious extension dysfunction after bleomycin chemotherapy. She was initially misdiagnosed with Dupuytren's disease clinically, and the definite diagnosis was finally confirmed by careful intraoperative exploration and postoperative pathological examination.

## Case report

**Figure 1:**
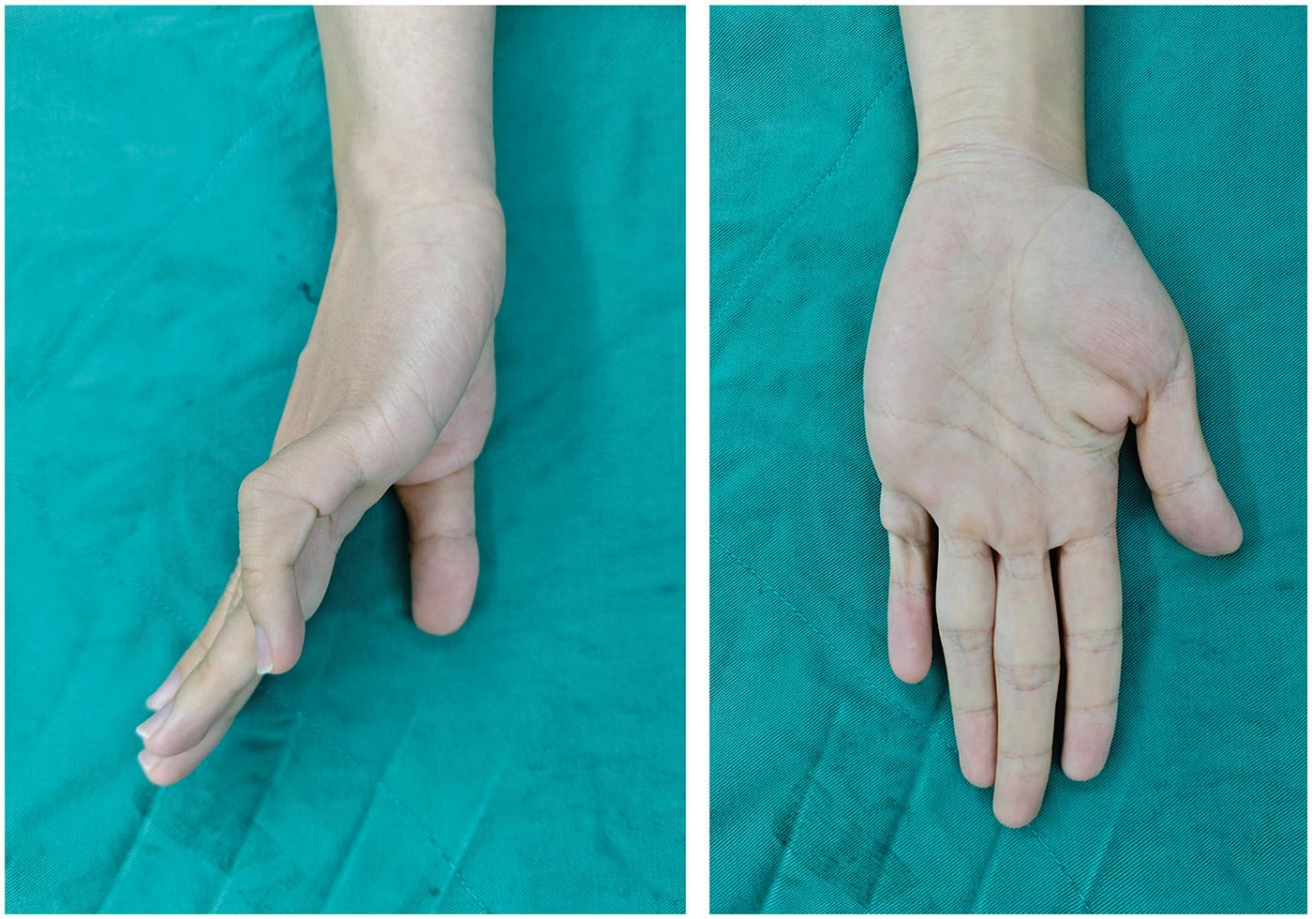
The patient demonstrates finger flexion and extension.

**Figure 2:**
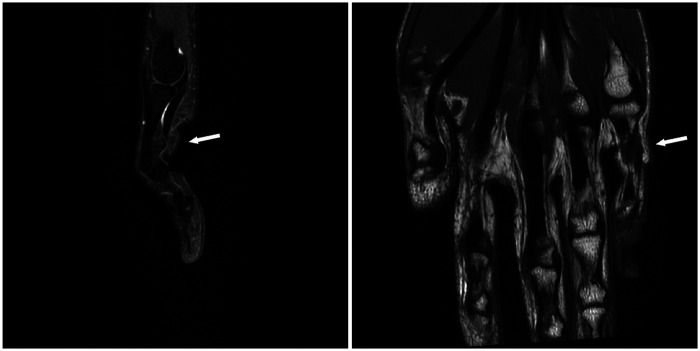
Preoperative MRI examination: sagittal and coronal images of the patient's left little finger.

A 26-year-old female patient presented with a 2-year history of flexion deformity of the left little finger. She complained of subcutaneous mass on the volar side of the proximal segment of the left little finger accompanied by limited flexion and extension movement. She had no local trauma history, no swelling and pain, no erythema or pruritus. She had no history of smoking and no family history of severe diseases. She had a 9-year medical history of Hodgkin's lymphoma (HL). Physical examination revealed flexion deformity of the proximal interphalangeal (PIP) joint of the left little finger, with a range of motion of 60°–110° (Figure 1). A hard nodule measuring approximately 0.4 cm × 0.3 cm was palpable subcutaneously. The range of motion of the metacarpophalangeal (MP) joint and distal interphalangeal joint was normal, together with intact blood circulation and sensation. The DASH score was 0.83. A suspicious subcutaneous mass was noticed in the left little finger in 2015. The patient was diagnosed with HL in 2016 and received bleomycin chemotherapy in the second half of the same year. Subcutaneous mass gradually appeared at the proximal segment of the left little finger starting from 2020. On August 25, 2021, color Doppler ultrasound showed: “A cystic nodule measuring about 0.4 cm × 0.3 cm was detected beside the flexor tendon of the proximal phalanx of the left little finger, with clear boundary, regular shape and good internal sound transmission. No obvious blood flow signal was observed on Color Doppler Flow Imaging (CDFI).” The diagnostic conclusion was ganglion cyst of the flexor tendon of the left little finger. The lesion disappeared after repeated extrusion but relapsed many times afterwards. In 2023, hard nodules were found in the skin of the left little finger, followed by gradual contracture and finger extension dysfunction. MRI examination performed on August 8, 2025 (Figure 2) revealed: “Irregular long T1 and short T2 signals with ill-defined margins were observed in the soft tissues adjacent to the flexor digitorum longus tendon at the level of the fifth proximal phalanx of the left hand, with a size of approximately 0.3 cm × 0.4 cm × 1.1 cm. A small amount of fluid signal was seen around the proximal interphalangeal joint and flexor tendons.” Accordingly, the preoperative diagnoses were formulated as: (1) Dupuytren's disease of the left little finger; (2) Ganglion cyst of the left little finger.

**Figure 3:**
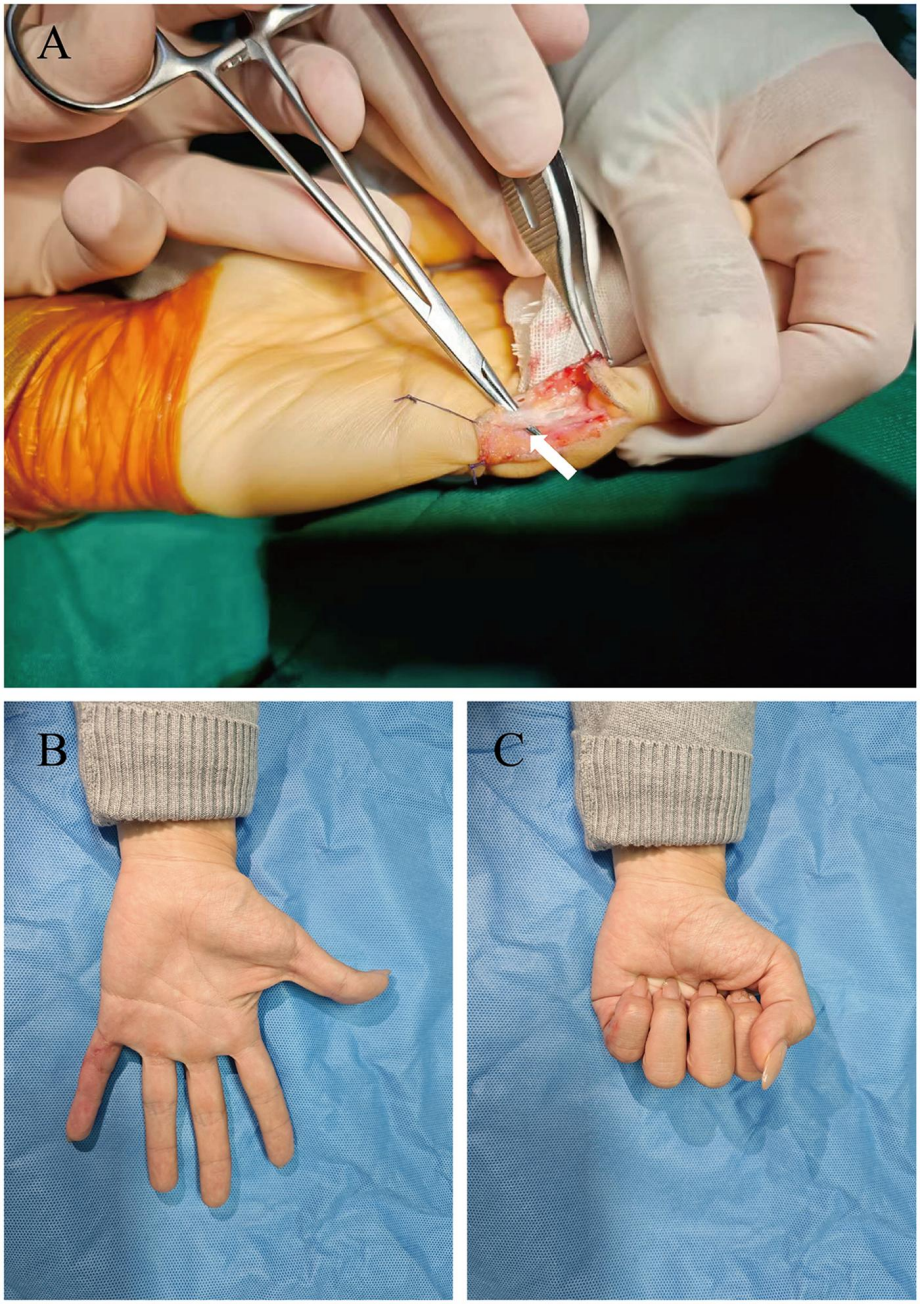
Intraoperative view and postoperative outcome of the left little finger. **(A)** Intraoperative scar location; **(B,C)** flexion and extension of the left little finger at 7 weeks postoperatively.

The operation was performed under brachial plexus block anesthesia. An oblique volar incision was made at the proximal segment of the left little finger and extended distally longitudinally along the ulnar side of the middle segment, with a total length of approximately 5 cm. After skin incision, the scar was located in the subcutaneous layer with punctate adhesions to the skin. The hard scar tissue distributed over the volar side of the proximal segment and the ulnar side of the middle segment (Figure 3A). After resection of subcutaneous scar tissue, a longitudinal cord-like structure was found on the surface of the ulnar neurovascular bundle in Zone Ⅱ, extending distally to distal Zone Ⅰ. 2160;. A hard nodule was observed at the central volar aspect of Zone Ⅱ. 2161;. The radial digital nerve was covered by scar tissue, and the proper ulnar digital nerve was completely encased in scars. The flexor tendon sheath remained intact without involvement. Both digital nerves and digital arteries on bilateral sides were sharply dissected and fully protected, followed by complete scar excision. Immediate passive motion examination after surgery showed that the range of motion of the proximal interphalangeal joint reached 0°–110°, the incision was tension-free, and full extension of the little finger was achieved. Intraoperative findings were consistent with the manifestation of Dupuytren's disease. The postoperative DASH score was 5.83 at 10 days after surgery. Seven weeks postoperatively, favorable active finger movement was achieved with a DASH score of 0 (Figures 3B,C). The detailed chronological sequence is presented in Table 1.

**Table 1:** Based on the timeline, clinical events are listed below.

Time	Events
2015	A suspicious subcutaneous mass was found in the left little finger
2016	Diagnosed with HL and received bleomycin chemotherapy
2020	Subcutaneous mass gradually developed at the proximal part of the left little finger
2021	Confirmed as ganglion cyst by ultrasonography
2023	Scar contracture occurred at the proximal left little finger with progressive flexion deformity
2025	The patient presented with 2-year flexion deformity of the left little finger and a DASH score of 0.83, which was preoperatively misdiagnosed as Dupuytren's disease
August 2025	Surgical treatment was performed for complete scar excision
7 weeks postoperatively	Full functional recovery and satisfactory active movement of the little finger were achieved, with a DASH score of 0

**Figure 4:**
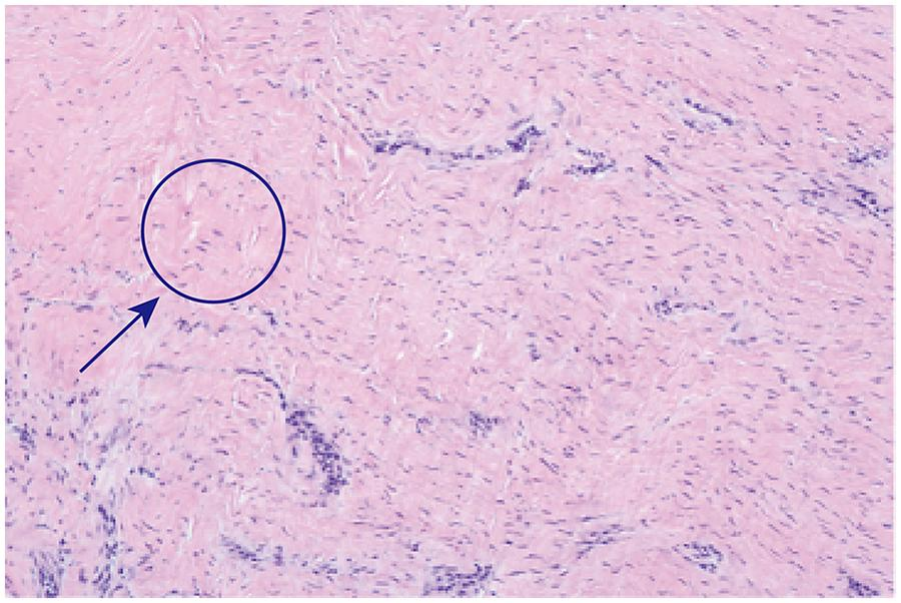
Histopathological image. Massively proliferated fibroblasts arranged in a dendritic pattern with abundant interstitial collagen. Dense collagen bundles are indicated by blue circles and arrows.

Surprisingly, preliminary pathological examination of the excised scar tissue specimens was performed (Figure 4), and the initial pathological diagnosis was subcutaneous scar tissue of the left little finger consistent with fibrous tumor of tendon sheath. Further immunohistochemical staining revealed hyperplasia of fibrous tissue, scar tissue and synovial tissue in the affected little finger, which was morphologically consistent with ruptured ganglion cyst complicated with fibrous hyperplasia and scar formation. The immunohistochemical results were as follows: SMA (+), CD34 (−), CD68 (−), membranous B-Catenin (+), and the Ki-67 positive rate was 5%. SMA positivity indicated proliferation of myofibroblasts. Negative CD34 ruled out the diagnosis of Dupuytren's disease. The low Ki-67 proliferative activity confirmed the benign nature of this fibroproliferative lesion. The final revised diagnosis was scar contracture secondary to ruptured ganglion cyst.

## Discussion

This is a case report of rare scar contracture secondary to ruptured GC that was misdiagnosed as Dupuytren's disease. A previous study found that spontaneous regression occurred in 20 out of 39 untreated patients with GC (7). In addition, a clinical study showed that spontaneous rupture occurred in 1 out of 12 patients with GC, and the symptoms were completely relieved after rupture in this case (8). The formation of scar contracture after GC rupture is a complex biological process, which is mainly jointly affected by multiple factors including tissue injury, inflammatory response, abnormal healing and unbalanced collagen metabolism (9, 10). This indicates that although GC is mostly a benign lesion, it presents diverse clinical outcomes. Rare complications such as scar contracture should also be alerted to avoid misdiagnosis and unnecessary interventions.

The initial diagnosis tended to be Dupuytren's disease mainly based on its typical clinical manifestations. The patient presented with progressive flexion deformity of the little finger, and hard subcutaneous nodules and cord-like structures extending from MP to PIP could be palpated on physical examination. These features were highly consistent with the classic signs of Dupuytren's disease. In addition, the patient had no history of trauma, acute infection or other conditions that may cause secondary contracture, which naturally directed the diagnostic thinking toward this common fibroproliferative disorder. Preoperative ultrasonography and MRI findings supported the diagnosis of GC. After GC rupture, the internal mucoid substance infiltrates into the surrounding subcutaneous tissues, acting as foreign bodies and triggering local chronic inflammatory responses and fibrous tissue proliferation. In certain individuals, especially those with a predisposition to fibrosis such as patients with a history of bleomycin chemotherapy in this case, this repair process becomes excessively active, resulting in massive collagen deposition and the formation of firm scar tissue. As the scar tissue matures and contracts, it pulls the adjacent soft tissues and skin, eventually leading to progressive flexion deformity of the finger joints. The final pathological diagnosis confirmed scar contracture secondary to ruptured GC. Combined with the patient's history of recurrent cysts, the complete clinical, imaging and pathological evidence chain fully elucidates the pathological progression from repeated cyst rupture and aberrant fibrosis to eventual scar contracture formation.

The mechanism of scar contracture formation following GC rupture is complicated. Firstly, cyst fluid escapes after GC rupture and causes direct mechanical damage to surrounding soft tissues, which activates the body's repair mechanism, induces abnormal proliferation of fibroblasts and excessive collagen deposition, and consequently leads to scar formation (9). Secondly, the release of cyst fluid triggers local aseptic inflammatory reactions (11). It recruits immune cells and promotes the secretion of various pro-inflammatory cytokines such as transforming growth factor-β1 (TGF-β1), which further accelerates the activation and proliferation of fibroblasts and myofibroblasts (12). As contractile functional cells, persistent contraction of myofibroblasts serves as one of the vital causes of scar contracture (13). Thirdly, disturbed or abnormal healing processes, including persistent inflammation, abnormal mechanical stress and genetic predisposition, may result in excessive collagen deposition and cross-linking, thus forming hypertrophic scars or scar contractures (12). Fourthly, imbalanced collagen metabolism constitutes the pathophysiological basis of scar contracture. The sustained existence and enhanced activity of myofibroblasts make the synthesis of collagens, especially type I and type III collagen, far exceed their degradation (10). Moreover, abnormal arrangement and cross-linking of collagen fibers contribute to the stiffness and contractility of scar tissue (14). Such excessive collagen deposition and structural rearrangement ultimately thicken and harden scar tissue and generate persistent contractile force, eventually resulting in contracture.

Scar contracture induced by GC rupture is extremely rare yet clinically possible, especially in this patient with Hodgkin's lymphoma who received bleomycin chemotherapy during treatment. Alpha-smooth muscle actin (α-SMA) is a biomarker of myofibroblasts. TGF-β1 and matrix stiffness can specifically upregulate the expression of α-SMA and cellular stiffness in fibroblasts of Dupuytren's disease, thereby contributing to progressive contracture (15). Fibroblasts derived from Dupuytren's lesions present high α-SMA expression, regularly arranged stress fibers and increased cellular stiffness, while normal fibroblasts and common scar fibroblasts show low or absent α-SMA expression with lower cellular stiffness. Although the present case exhibited positive SMA expression and shared similar clinical and biomechanical features with Dupuytren's disease, negative CD34 expression ruled out classic Dupuytren's disease. This finding suggests that bleomycin may act as an inducing factor for fibroblast transdifferentiation after GC rupture. Previous studies have confirmed that bleomycin can upregulate the mRNA expression of type I collagen, fibronectin and decorin in human dermal fibroblasts, and promote the expression of pro-fibrotic cytokines including transforming growth factor-β (TGF-β) and connective tissue growth factor (CTGF) (16). Imbalanced regulation of TGF-β1 leads to continuous collagen accumulation, converting temporary scar tissue into progressive lesions and even triggering organ fibrosis (17). As a widely used chemotherapeutic agent for various malignant tumors, bleomycin can also cause rare characteristic flagellate dermatitis (18). Local skin inflammation and minor trauma caused by scratching may further induce spontaneous scar hyperplasia. Nevertheless, the patient had no clinical manifestations such as skin erythema, pruritus or typical flagellate dermatitis throughout the disease course, which excludes the possibility that bleomycin induces local scar formation via flagellate dermatitis. Further investigations are still needed to clarify whether bleomycin participates in the occurrence and progression of scar lesions in this case through other potential pathological mechanisms.

It is usually difficult to confirm the diagnosis of scar contracture caused by GC rupture before pathological examination, mainly due to the absence of specific clinical manifestations and imaging features. In this case, subcutaneous mass gradually emerged at the proximal left little finger for nearly 4 years after bleomycin chemotherapy. Intraoperatively, cord-like scar tissue was found beneath the finger skin, accompanied by flexion contracture of MP and PIP joints. Accordingly, we initially diagnosed the finger deformity as Dupuytren's disease, which led to misdiagnosis. The optimal therapeutic strategy is complete resection of scar contracture with clear surgical margins. Although scar contracture secondary to GC rupture is a rare clinical entity, clinicians should include it in the differential diagnosis to avoid unnecessary aggressive treatment. For indistinct soft tissue proliferative lesions in clinical practice, standardized immunohistochemical panels are recommended to distinguish Dupuytren's disease, fibromatosis and other subcutaneous proliferative disorders. For difficult cases, pathological examination remains the gold standard for definitive diagnosis.

This case firstly fully demonstrates the whole process of scar contracture induced by ruptured GC being misdiagnosed as Dupuytren's disease, providing a replicable reference for differential diagnosis and surgical management in hand surgery. Nevertheless, as a single case study, it lacks molecular experimental evidence and long-term follow-up data, which limits the confirmation of causal relationship and generalizability of the conclusions. Further in-depth researches are required in the future. This case indicates that subcutaneous scar contracture has diverse etiologies. Clinical diagnosis requires multi-dimensional comprehensive analysis combined with detailed medical history, special medication history, imaging evaluation and definitive pathological evidence to prevent misdiagnosis and inappropriate treatment. Complete surgical excision of scar tissue and release of contracture is a reliable therapeutic approach with favorable patient prognosis. Emphasis on comprehensive judgment and accurate diagnosis helps avoid misjudging such rare disorders as common diseases and thereby prevent unnecessary overtreatment.

## Conclusion

Combined with the clinical data, imaging findings, immunohistochemical results and history of special medication of this case, the patient's age and gender at onset were inconsistent with the high-incidence characteristics of typical Dupuytren's disease. Meanwhile, we found that the profibrotic effect of bleomycin combined with local chronic inflammation after GC rupture may jointly lead to this secondary scar contracture. In clinical practice, hand lesions with similar deformities should not be diagnosed merely based on physical signs, and special factors such as medication history and history of cyst rupture need to be comprehensively investigated. This case provides a new perspective for the diagnosis of scar contracture caused by hand GC rupture, and emphasizes that meticulous intraoperative exploration and postoperative pathological verification are essential for atypical cases. Accurate diagnosis is the prerequisite for appropriate treatment. In this case, complete scar resection successfully relieved contracture and restored hand function, avoiding long-term or excessive treatment resulting from misdiagnosing rare conditions as common diseases.

## Patient perspective

During the outpatient follow-up at 7 weeks postoperatively, the flexion contracture deformity of the affected finger was completely corrected, and the little finger could be fully extended with obviously improved basic motor function compared with preoperative status. The patient stated that it was the first time for many years to complete the waving movement smoothly with all five fingers closed together. Long-term follow-up at 9 months after surgery revealed that the flexion and extension range of motion of the affected finger returned to normal. Fine hand functions such as grasping and pinching were flexible and unobstructed. The cutaneous sensation of the finger was normal without numbness, pain, swelling or other discomforts, and no local recurrence was observed at the lesion site. The postoperative scar was inconspicuous, exerting no obvious influence on daily life and appearance. The patient was highly satisfied with the therapeutic effect and functional recovery of the finger.

## Data Availability

The original contributions presented in the study are included in the article/Supplementary Material, further inquiries can be directed to the corresponding author.
